# *catena*-Poly[[[aqua­copper(II)]-bis­[μ-*N*,*N*′-bis­(pyridin-4-yl)isophthalamide]-[aqua­copper(II)]-di-μ-sulfato] dimethyl­formamide disolvate]

**DOI:** 10.1107/S1600536813003413

**Published:** 2013-02-16

**Authors:** Er-Peng Zhang, Li-Ping Wang, Yu-Fei Wang

**Affiliations:** a Henan Center for Disease Control and Prevention, Zhengzhou 450016, People’s Republic of China; b College of Chemical and Food Engineering, ZhongZhou University, Zhengzhou 450044, People’s Republic of China

## Abstract

In the title coordination polymer, {[Cu(SO_4_)(C_18_H_14_N_4_O_2_)(H_2_O)]·C_3_H_7_NO}_*n*_, the Cu^II^ ion is coordinated by two N atoms of two briding *N*,*N*′-bis­(pyridin-4-yl)isophthalamide ligands, two O atoms of two bridging SO_4_^2−^ anions and a water mol­ecule, giving a distorted CuN_2_O_3_ square-pyramidal geometry. The whole repeating mol­ecular unit is generated by inversion symmetry. This leads to the formation of a looped-chain one-dimensional coordination polymer propagating along [110]. The dimethyl­formamide (DMF) mol­ecules are linked to the chains *via* O—H⋯O hydrogen bonds. The chains are linked *via* N—H⋯O hydrogen bonds, forming two-dimensional networks parallel to (001). There are also a number of C—H⋯O inter­actions present and a parallel slipped π–π inter­action. The latter involves inversion-related pyridine rings with a centroid–centroid distance of 3.594 (2) Å [normal distance = 3.3338 (13) and slippage = 1.341 Å]. These inter­actions lead to the formation of a three-dimensional structure.

## Related literature

For background to metal complexes with a *N,N′*-bis-(4-pyrid­yl)isophthalamide ligand, see: Adarsh *et al.* (2009 ▶); Gong *et al.* (2010 ▶, 2011 ▶); Kim *et al.* (2011 ▶).
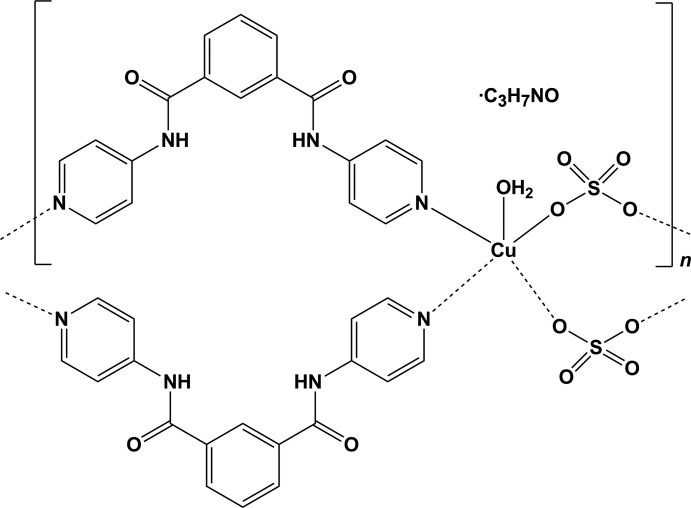


## Experimental

### 

#### Crystal data

[Cu(SO_4_)(C_18_H_14_N_4_O_2_)(H_2_O)]·C_3_H_7_NO
*M* *_r_* = 569.06
Triclinic, 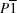

*a* = 10.389 (2) Å
*b* = 11.092 (1) Å
*c* = 12.105 (2) Å
α = 63.47 (3)°
β = 79.75 (2)°
γ = 71.08 (3)°
*V* = 1179.8 (4) Å^3^
*Z* = 2
Mo *K*α radiation
μ = 1.07 mm^−1^
*T* = 293 K
0.28 × 0.24 × 0.20 mm

#### Data collection

Rigaku Saturn 724 diffractometer
Absorption correction: multi-scan (*SADABS*; Sheldrick, 1996 ▶) *T*_min_ = 0.753, *T*_max_ = 0.814
14785 measured reflections
5581 independent reflections
4622 reflections with *I* > 2σ(*I*)
*R*_int_ = 0.039

#### Refinement

*R*[*F* ^2^ > 2σ(*F* ^2^)] = 0.052
*wR*(*F* ^2^) = 0.124
*S* = 1.07
5581 reflections
339 parameters
4 restraints
H atoms treated by a mixture of independent and constrained refinement
Δρ_max_ = 0.44 e Å^−3^
Δρ_min_ = −0.48 e Å^−3^

### 

Data collection: *CrystalClear* (Rigaku/MSC, 2006 ▶); cell refinement: *CrystalClear*; data reduction: *CrystalClear*; program(s) used to solve structure: *SHELXS97* (Sheldrick, 2008 ▶); program(s) used to refine structure: *SHELXL97* (Sheldrick, 2008 ▶); molecular graphics: *SHELXTL* (Sheldrick, 2008 ▶); software used to prepare material for publication: *publCIF* (Westrip, 2010 ▶).

## Supplementary Material

Crystal structure: contains datablock(s) I, global. DOI: 10.1107/S1600536813003413/su2555sup1.cif

Structure factors: contains datablock(s) I. DOI: 10.1107/S1600536813003413/su2555Isup2.hkl

Additional supplementary materials:  crystallographic information; 3D view; checkCIF report

## Figures and Tables

**Table 1:** Hydrogen-bond geometry (Å, °)

*D*—H⋯*A*	*D*—H	H⋯*A*	*D*⋯*A*	*D*—H⋯*A*
N3—H3*A*⋯O2^i^	0.86 (3)	2.03 (3)	2.867 (3)	164 (3)
N4—H4*A*⋯O3^i^	0.87 (3)	2.25 (3)	3.103 (4)	168 (3)
O5—H5*A*⋯O8^ii^	0.82 (3)	1.81 (3)	2.626 (4)	179 (4)
O5—H5*B*⋯O2	0.81 (3)	1.90 (3)	2.684 (3)	164 (4)
C4—H4⋯O8	0.93	2.45	3.325 (4)	157
C18—H18⋯O6^iii^	0.93	2.55	3.277 (4)	136
C19—H19⋯O4^iv^	0.93	2.47	3.286 (4)	146
C20—H20*C*⋯O3^v^	0.96	2.58	3.226 (6)	125

Symmetry codes: (i) 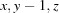; (ii) 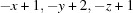; (iii) 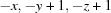; (iv) 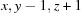; (v) 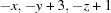.

**Fig. 1.**
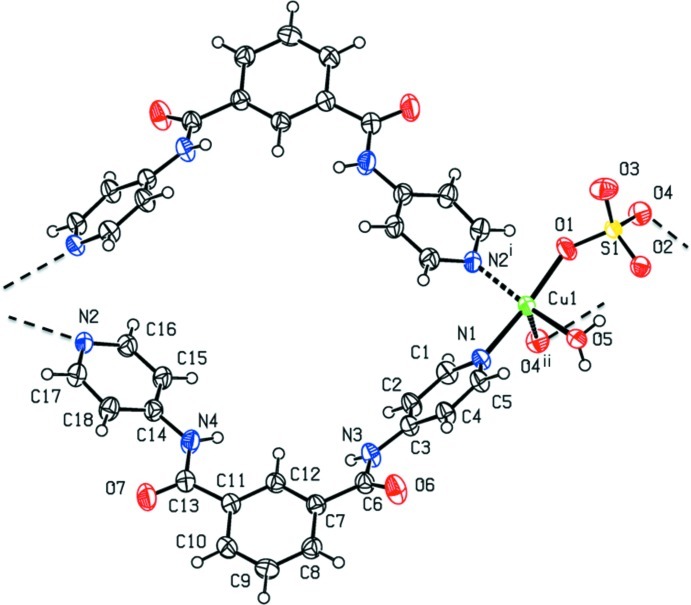
A fragment of the title complex showing the atom labelling. The displacement ellipsoids are drawn at the 30% probability level. H atoms and solvent molecules have been omitted for clarity [symmetry codes: (i) -x, -y+2, -z; (ii) -x+1, -y+3, -z].

**Fig. 2.**
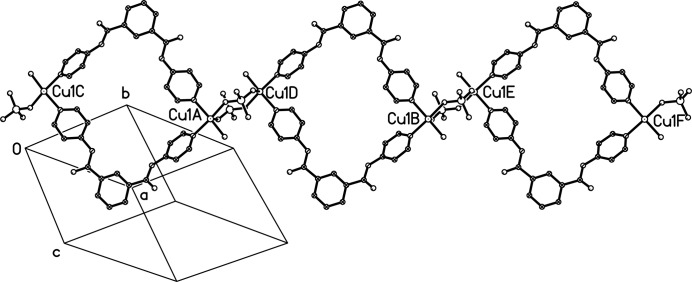
View of the extended one-dimensional looped-chain structure of the title complex.

## supplementary crystallographic information

### Comment

The bis-pyridyl-bis-amide ligands have been used to construct various metal-organic frameworks (MOFs), not only due to their conformational flexibility but also due to the multiple hydrogen bonding sites in the ligand backbone (Adarsh *et al.*, 2009; Gong *et al.*, 2010, 2011; Kim *et al.*, 2011). In this work, the bis-pyridyl-bis-amide ligand, *N,N'*-bis-(4-pyridyl)isophthalamide (bppa), has been used to generate the title coordination polymer whose crystal structure is reported on herein.

The coordination enviroment of the Cu^II^ center in the title complex is shown in Fig. 1. In the distorted square pyramidal geometry of the Cu^II^ ion the basal positions are occupied by two pyridyl N atoms of briding bppa ligands (N1 and N2^i^), an O atom (O1) of a briding SO_4_^2-^ anion and the O atom (O5) of a coordinated water molecule, while the axial position is occupied by the O atom (O4^ii^) of a second briding SO_4_^2-^ anion. The Cu^II^ ions are connected by a pair of bridging SO_4_^2-^ anions, yielding a centrosymmetric Cu_2_(SO_4_)_2_ binuclear unit with a Cu···Cu distance of 4.772( ) Å. The binuclear units are further linked by two bppa ligands to give a looped-chain coordination polymer extending along [1 1 0], as shown in Fig. 2. The distance between two Cu^II^ ions bridged by the bppa ligands is ca. 13.87 Å.

In the crystal, the chains are linked via N-H···O to form two-dimensional networks extending in the a and b directions. The dimethyl formamide (DMF) molecules are linked to the chains via O-H···O hydrogen bonds (Table 1). There are also a number of C-H···O interactions present and a parallel slipped π-π interaction. The latter involves inversion related N2/C14-C18 pyridine rings with a centroid-to-centroid distance 3.594 (2) Å [normal distance 3.3338 (13) Å, slippage 1.341 Å]. These interactions lead to the formatin of a three-diemnsional structure.

### Experimental

The ligand *N,N'*-bis-(4-pyridyl)isophthalamide (0.03 mmol, 10 mg) in DMF (5 ml) was added dropwise to a methanol solution (5 ml) of CuSO_4_^.^5H_2_O; (0.03 mmol, 7.5 mg) in methanol. The resulting solution was allowed to stand at room temperature. After one week good quality blue crystals were obtained.

### Refinement

The NH and water OH H-atoms were located from difference Fourier maps and refined with U_iso_(H) = 1.2U_eq_(N) and = 1.5U_eq_(O). The C-bound H-atoms were placed in calculated positions and treated as riding atoms: C—H = 0.93 Å (aromatic) and 0.96 Å (methyl), with U_iso_(H) = 1.2U_eq_(C-aromatic) and = 1.5U_eq_(C methyl).

### Crystal data

**Table:**

[Cu(SO_4_)(C_18_H_14_N_4_O_2_)(H_2_O)]·C_3_H_7_NO	*Z* = 2
*M**_r_* = 569.06	*F*(000) = 586
Triclinic, *P*1	*D*_x_ = 1.602 Mg m^−^^3^
Hall symbol: -P 1	Mo *K*α radiation, λ = 0.71073 Å
*a* = 10.389 (2) Å	Cell parameters from 2540 reflections
*b* = 11.092 (1) Å	θ = 1.3–25.5°
*c* = 12.105 (2) Å	µ = 1.07 mm^−^^1^
α = 63.47 (3)°	*T* = 293 K
β = 79.75 (2)°	Prism, blue
γ = 71.08 (3)°	0.28 × 0.24 × 0.20 mm
*V* = 1179.8 (4) Å^3^	

### Data collection

**Table:**

Rigaku Saturn 724 diffractometer	5581 independent reflections
Radiation source: fine-focus sealed tube	4622 reflections with *I* > 2σ(*I*)
Graphite monochromator	*R*_int_ = 0.039
Detector resolution: 28.5714 pixels mm^-1^	θ_max_ = 27.9°, θ_min_ = 2.5°
ω scans	*h* = −13→13
Absorption correction: multi-scan (*SADABS*; Sheldrick, 1996)	*k* = −14→14
*T*_min_ = 0.753, *T*_max_ = 0.814	*l* = −15→15
14785 measured reflections	

### Refinement

**Table:**

Refinement on *F*^2^	Primary atom site location: structure-invariant direct methods
Least-squares matrix: full	Secondary atom site location: difference Fourier map
*R*[*F*^2^ > 2σ(*F*^2^)] = 0.052	Hydrogen site location: inferred from neighbouring sites
*wR*(*F*^2^) = 0.124	H atoms treated by a mixture of independent and constrained refinement
*S* = 1.07	*w* = 1/[σ^2^(*F*_o_^2^) + (0.0562*P*)^2^ + 0.3078*P*] where *P* = (*F*_o_^2^ + 2*F*_c_^2^)/3
5581 reflections	(Δ/σ)_max_ = 0.001
339 parameters	Δρ_max_ = 0.44 e Å^−^^3^
4 restraints	Δρ_min_ = −0.48 e Å^−^^3^

### Special details

**Table:**

Geometry. All e.s.d.'s (except the e.s.d. in the dihedral angle between two l.s. planes) are estimated using the full covariance matrix. The cell e.s.d.'s are taken into account individually in the estimation of e.s.d.'s in distances, angles and torsion angles; correlations between e.s.d.'s in cell parameters are only used when they are defined by crystal symmetry. An approximate (isotropic) treatment of cell e.s.d.'s is used for estimating e.s.d.'s involving l.s. planes.
Refinement. Refinement of *F*^2^ against ALL reflections. The weighted *R*-factor *wR* and goodness of fit *S* are based on *F*^2^, conventional *R*-factors *R* are based on *F*, with *F* set to zero for negative *F*^2^. The threshold expression of *F*^2^ > σ(*F*^2^) is used only for calculating *R*-factors(gt) *etc*. and is not relevant to the choice of reflections for refinement. *R*-factors based on *F*^2^ are statistically about twice as large as those based on *F*, and *R*- factors based on ALL data will be even larger.

### Fractional atomic coordinates and isotropic or equivalent isotropic displacement parameters (Å^2^)

**Table:**

	*x*	*y*	*z*	*U*_iso_*/*U*_eq_	
Cu1	0.38105 (3)	1.33009 (3)	0.12726 (3)	0.02904 (12)	
N1	0.3870 (2)	1.1263 (2)	0.2105 (2)	0.0306 (5)	
N2	−0.2404 (2)	0.6278 (2)	−0.0103 (2)	0.0318 (5)	
N3	0.3422 (3)	0.7212 (3)	0.3637 (2)	0.0346 (6)	
H3A	0.353 (3)	0.686 (3)	0.311 (2)	0.041*	
N4	0.0555 (3)	0.5102 (3)	0.2416 (2)	0.0390 (6)	
H4A	0.111 (3)	0.562 (3)	0.214 (3)	0.047*	
N5	0.0872 (3)	0.9732 (3)	0.7526 (3)	0.0567 (8)	
O1	0.3441 (3)	1.5323 (2)	0.0619 (2)	0.0509 (6)	
O2	0.4349 (2)	1.5789 (2)	0.20428 (18)	0.0343 (5)	
O3	0.2229 (2)	1.7265 (2)	0.1090 (2)	0.0485 (6)	
O4	0.4322 (2)	1.7294 (2)	−0.01280 (18)	0.0394 (5)	
O5	0.4686 (2)	1.3056 (2)	0.2695 (2)	0.0360 (5)	
H5A	0.5446 (18)	1.251 (3)	0.284 (3)	0.054*	
H5B	0.474 (4)	1.3836 (18)	0.251 (3)	0.054*	
O6	0.3448 (3)	0.6518 (2)	0.57191 (19)	0.0510 (6)	
O7	0.0307 (3)	0.2996 (3)	0.3841 (3)	0.0766 (10)	
O8	0.2884 (3)	0.8735 (3)	0.6849 (3)	0.0816 (10)	
C1	0.4035 (3)	1.0521 (3)	0.1435 (3)	0.0350 (7)	
H1	0.4239	1.0942	0.0593	0.042*	
C2	0.3919 (3)	0.9193 (3)	0.1921 (3)	0.0350 (7)	
H2	0.4033	0.8731	0.1416	0.042*	
C3	0.3626 (3)	0.8524 (3)	0.3190 (3)	0.0301 (6)	
C4	0.3509 (3)	0.9258 (3)	0.3902 (3)	0.0343 (6)	
H4	0.3347	0.8849	0.4753	0.041*	
C5	0.3638 (3)	1.0592 (3)	0.3320 (3)	0.0342 (6)	
H5	0.3559	1.1069	0.3805	0.041*	
C6	0.3258 (3)	0.6324 (3)	0.4860 (3)	0.0332 (6)	
C7	0.2840 (3)	0.5079 (3)	0.5039 (3)	0.0308 (6)	
C8	0.3220 (3)	0.3856 (3)	0.6112 (3)	0.0357 (7)	
H8	0.3719	0.3824	0.6696	0.043*	
C9	0.2842 (4)	0.2693 (3)	0.6295 (3)	0.0475 (8)	
H9	0.3099	0.1869	0.7003	0.057*	
C10	0.2090 (3)	0.2740 (3)	0.5440 (3)	0.0440 (8)	
H10	0.1854	0.1944	0.5572	0.053*	
C11	0.1680 (3)	0.3970 (3)	0.4378 (3)	0.0341 (6)	
C12	0.2061 (3)	0.5133 (3)	0.4189 (3)	0.0313 (6)	
H12	0.1793	0.5960	0.3485	0.038*	
C13	0.0795 (3)	0.3964 (3)	0.3530 (3)	0.0409 (7)	
C14	−0.0426 (3)	0.5437 (3)	0.1590 (3)	0.0340 (6)	
C15	−0.0365 (3)	0.6471 (3)	0.0409 (3)	0.0376 (7)	
H15	0.0330	0.6907	0.0166	0.045*	
C16	−0.1348 (3)	0.6846 (3)	−0.0406 (3)	0.0382 (7)	
H16	−0.1282	0.7524	−0.1204	0.046*	
C17	−0.2423 (3)	0.5272 (3)	0.1045 (3)	0.0369 (7)	
H17	−0.3129	0.4853	0.1275	0.044*	
C18	−0.1476 (3)	0.4817 (3)	0.1902 (3)	0.0370 (7)	
H18	−0.1538	0.4104	0.2682	0.044*	
C19	0.2186 (4)	0.9110 (4)	0.7613 (3)	0.0545 (9)	
H19	0.2605	0.8947	0.8304	0.065*	
C20	0.0111 (6)	1.0277 (7)	0.8395 (5)	0.117 (2)	
H20A	0.0717	1.0157	0.8972	0.175*	
H20B	−0.0559	0.9783	0.8832	0.175*	
H20C	−0.0336	1.1257	0.7962	0.175*	
C21	0.0159 (4)	0.9925 (5)	0.6498 (4)	0.0822 (14)	
H21A	0.0778	0.9484	0.6009	0.123*	
H21B	−0.0188	1.0909	0.5994	0.123*	
H21C	−0.0583	0.9509	0.6814	0.123*	
S1	0.35897 (7)	1.64365 (7)	0.09048 (6)	0.02617 (16)	

### Atomic displacement parameters (Å^2^)

**Table:**

	*U*^11^	*U*^22^	*U*^33^	*U*^12^	*U*^13^	*U*^23^
Cu1	0.0327 (2)	0.0243 (2)	0.0321 (2)	−0.01067 (15)	−0.00462 (14)	−0.01040 (15)
N1	0.0385 (13)	0.0254 (12)	0.0305 (12)	−0.0126 (10)	−0.0007 (10)	−0.0117 (10)
N2	0.0321 (12)	0.0323 (13)	0.0355 (13)	−0.0133 (10)	−0.0032 (10)	−0.0142 (11)
N3	0.0520 (16)	0.0320 (13)	0.0266 (12)	−0.0217 (12)	0.0028 (11)	−0.0129 (11)
N4	0.0379 (14)	0.0442 (16)	0.0385 (14)	−0.0215 (12)	−0.0046 (11)	−0.0124 (12)
N5	0.0508 (18)	0.0533 (19)	0.0501 (18)	−0.0023 (15)	0.0016 (14)	−0.0175 (15)
O1	0.0801 (18)	0.0271 (11)	0.0528 (14)	−0.0155 (11)	−0.0306 (13)	−0.0125 (10)
O2	0.0414 (12)	0.0321 (11)	0.0329 (11)	−0.0089 (9)	−0.0065 (9)	−0.0155 (9)
O3	0.0326 (12)	0.0469 (14)	0.0551 (14)	0.0003 (10)	0.0030 (10)	−0.0214 (12)
O4	0.0353 (11)	0.0422 (12)	0.0366 (11)	−0.0174 (10)	0.0062 (9)	−0.0111 (10)
O5	0.0409 (12)	0.0268 (11)	0.0405 (12)	−0.0070 (9)	−0.0109 (10)	−0.0126 (10)
O6	0.0858 (19)	0.0503 (14)	0.0287 (11)	−0.0387 (13)	−0.0043 (11)	−0.0123 (10)
O7	0.101 (2)	0.0432 (15)	0.088 (2)	−0.0383 (15)	−0.0528 (18)	0.0010 (14)
O8	0.0632 (18)	0.101 (2)	0.0707 (19)	0.0272 (17)	−0.0212 (15)	−0.0551 (19)
C1	0.0449 (17)	0.0358 (16)	0.0266 (14)	−0.0145 (14)	0.0023 (12)	−0.0142 (13)
C2	0.0505 (18)	0.0317 (15)	0.0296 (14)	−0.0172 (14)	0.0029 (13)	−0.0163 (13)
C3	0.0349 (15)	0.0281 (14)	0.0304 (14)	−0.0122 (12)	−0.0009 (12)	−0.0126 (12)
C4	0.0474 (18)	0.0330 (16)	0.0280 (14)	−0.0186 (14)	0.0027 (12)	−0.0137 (12)
C5	0.0434 (17)	0.0301 (15)	0.0346 (15)	−0.0144 (13)	0.0007 (13)	−0.0164 (13)
C6	0.0380 (16)	0.0323 (15)	0.0303 (14)	−0.0159 (13)	−0.0002 (12)	−0.0103 (12)
C7	0.0335 (15)	0.0302 (15)	0.0295 (14)	−0.0116 (12)	0.0003 (11)	−0.0118 (12)
C8	0.0388 (16)	0.0371 (16)	0.0306 (15)	−0.0140 (13)	−0.0059 (12)	−0.0098 (13)
C9	0.056 (2)	0.0301 (17)	0.0439 (18)	−0.0130 (15)	−0.0169 (16)	0.0015 (14)
C10	0.0509 (19)	0.0300 (16)	0.0484 (19)	−0.0170 (14)	−0.0113 (15)	−0.0068 (14)
C11	0.0344 (15)	0.0295 (15)	0.0387 (16)	−0.0122 (12)	−0.0035 (12)	−0.0114 (13)
C12	0.0357 (15)	0.0287 (15)	0.0284 (14)	−0.0120 (12)	−0.0021 (12)	−0.0085 (12)
C13	0.0429 (18)	0.0350 (17)	0.0471 (18)	−0.0144 (14)	−0.0104 (14)	−0.0135 (15)
C14	0.0342 (15)	0.0360 (16)	0.0387 (16)	−0.0099 (13)	−0.0029 (12)	−0.0209 (14)
C15	0.0337 (15)	0.0378 (17)	0.0423 (17)	−0.0183 (13)	−0.0019 (13)	−0.0118 (14)
C16	0.0402 (17)	0.0351 (17)	0.0388 (16)	−0.0169 (14)	−0.0043 (13)	−0.0095 (13)
C17	0.0352 (16)	0.0431 (18)	0.0370 (16)	−0.0168 (14)	−0.0009 (13)	−0.0168 (14)
C18	0.0359 (16)	0.0463 (18)	0.0323 (15)	−0.0185 (14)	0.0009 (12)	−0.0153 (14)
C19	0.055 (2)	0.053 (2)	0.044 (2)	0.0042 (18)	−0.0135 (17)	−0.0192 (18)
C20	0.101 (4)	0.128 (5)	0.106 (4)	−0.004 (4)	0.036 (4)	−0.069 (4)
C21	0.054 (3)	0.087 (3)	0.094 (4)	−0.019 (2)	−0.022 (2)	−0.021 (3)
S1	0.0283 (3)	0.0230 (3)	0.0297 (3)	−0.0089 (3)	−0.0005 (3)	−0.0122 (3)

### Geometric parameters (Å, º)

**Table:**

Cu1—O1	1.942 (2)	C2—H2	0.9300
Cu1—O5	1.961 (2)	C3—C4	1.395 (4)
Cu1—N1	2.007 (2)	C4—C5	1.367 (4)
Cu1—N2^i^	2.015 (2)	C4—H4	0.9300
Cu1—O4^ii^	2.269 (2)	C5—H5	0.9300
N1—C5	1.337 (3)	C6—C7	1.494 (4)
N1—C1	1.349 (3)	C7—C12	1.389 (4)
N2—C17	1.340 (4)	C7—C8	1.394 (4)
N2—C16	1.354 (4)	C8—C9	1.382 (4)
N2—Cu1^i^	2.015 (2)	C8—H8	0.9300
N3—C6	1.381 (3)	C9—C10	1.377 (4)
N3—C3	1.382 (3)	C9—H9	0.9300
N3—H3A	0.86 (3)	C10—C11	1.396 (4)
N4—C13	1.367 (4)	C10—H10	0.9300
N4—C14	1.401 (4)	C11—C12	1.382 (4)
N4—H4A	0.87 (3)	C11—C13	1.497 (4)
N5—C19	1.314 (5)	C12—H12	0.9300
N5—C20	1.435 (5)	C14—C15	1.383 (4)
N5—C21	1.460 (5)	C14—C18	1.384 (4)
O1—S1	1.481 (2)	C15—C16	1.380 (4)
O2—S1	1.473 (2)	C15—H15	0.9300
O3—S1	1.457 (2)	C16—H16	0.9300
O4—S1	1.454 (2)	C17—C18	1.369 (4)
O4—Cu1^ii^	2.269 (2)	C17—H17	0.9300
O5—H5A	0.82 (3)	C18—H18	0.9300
O5—H5B	0.81 (3)	C19—H19	0.9300
O6—C6	1.211 (3)	C20—H20A	0.9600
O7—C13	1.215 (4)	C20—H20B	0.9600
O8—C19	1.209 (4)	C20—H20C	0.9600
C1—C2	1.359 (4)	C21—H21A	0.9600
C1—H1	0.9300	C21—H21B	0.9600
C2—C3	1.401 (4)	C21—H21C	0.9600
			
O1—Cu1—O5	90.75 (9)	C7—C8—H8	120.5
O1—Cu1—N1	169.85 (10)	C10—C9—C8	120.8 (3)
O5—Cu1—N1	89.23 (10)	C10—C9—H9	119.6
O1—Cu1—N2^i^	85.21 (10)	C8—C9—H9	119.6
O5—Cu1—N2^i^	162.72 (10)	C9—C10—C11	120.6 (3)
N1—Cu1—N2^i^	91.82 (10)	C9—C10—H10	119.7
O1—Cu1—O4^ii^	102.68 (10)	C11—C10—H10	119.7
O5—Cu1—O4^ii^	100.05 (9)	C12—C11—C10	118.8 (3)
N1—Cu1—O4^ii^	87.31 (9)	C12—C11—C13	123.7 (3)
N2^i^—Cu1—O4^ii^	97.23 (9)	C10—C11—C13	117.5 (3)
C5—N1—C1	116.0 (2)	C11—C12—C7	120.6 (3)
C5—N1—Cu1	123.07 (19)	C11—C12—H12	119.7
C1—N1—Cu1	120.74 (19)	C7—C12—H12	119.7
C17—N2—C16	115.7 (3)	O7—C13—N4	122.5 (3)
C17—N2—Cu1^i^	118.66 (19)	O7—C13—C11	120.7 (3)
C16—N2—Cu1^i^	125.3 (2)	N4—C13—C11	116.7 (3)
C6—N3—C3	126.9 (2)	C15—C14—C18	118.2 (3)
C6—N3—H3A	116 (2)	C15—C14—N4	118.3 (3)
C3—N3—H3A	116 (2)	C18—C14—N4	123.5 (3)
C13—N4—C14	126.0 (3)	C16—C15—C14	119.1 (3)
C13—N4—H4A	119 (2)	C16—C15—H15	120.4
C14—N4—H4A	115 (2)	C14—C15—H15	120.4
C19—N5—C20	121.9 (4)	N2—C16—C15	123.4 (3)
C19—N5—C21	119.8 (3)	N2—C16—H16	118.3
C20—N5—C21	118.3 (4)	C15—C16—H16	118.3
S1—O1—Cu1	141.81 (14)	N2—C17—C18	124.6 (3)
S1—O4—Cu1^ii^	131.29 (13)	N2—C17—H17	117.7
Cu1—O5—H5A	116 (3)	C18—C17—H17	117.7
Cu1—O5—H5B	103 (3)	C17—C18—C14	118.8 (3)
H5A—O5—H5B	108 (4)	C17—C18—H18	120.6
N1—C1—C2	123.8 (3)	C14—C18—H18	120.6
N1—C1—H1	118.1	O8—C19—N5	123.9 (4)
C2—C1—H1	118.1	O8—C19—H19	118.0
C1—C2—C3	119.5 (3)	N5—C19—H19	118.0
C1—C2—H2	120.3	N5—C20—H20A	109.5
C3—C2—H2	120.3	N5—C20—H20B	109.5
N3—C3—C4	124.7 (3)	H20A—C20—H20B	109.5
N3—C3—C2	117.8 (2)	N5—C20—H20C	109.5
C4—C3—C2	117.4 (3)	H20A—C20—H20C	109.5
C5—C4—C3	118.4 (3)	H20B—C20—H20C	109.5
C5—C4—H4	120.8	N5—C21—H21A	109.5
C3—C4—H4	120.8	N5—C21—H21B	109.5
N1—C5—C4	124.9 (3)	H21A—C21—H21B	109.5
N1—C5—H5	117.5	N5—C21—H21C	109.5
C4—C5—H5	117.5	H21A—C21—H21C	109.5
O6—C6—N3	123.6 (3)	H21B—C21—H21C	109.5
O6—C6—C7	122.4 (3)	O4—S1—O3	111.13 (14)
N3—C6—C7	114.1 (2)	O4—S1—O2	110.28 (13)
C12—C7—C8	120.2 (3)	O3—S1—O2	109.69 (13)
C12—C7—C6	121.7 (3)	O4—S1—O1	108.42 (14)
C8—C7—C6	118.1 (3)	O3—S1—O1	107.80 (15)
C9—C8—C7	119.0 (3)	O2—S1—O1	109.46 (12)
C9—C8—H8	120.5		
			
O1—Cu1—N1—C5	−51.2 (6)	C8—C9—C10—C11	−0.8 (5)
O5—Cu1—N1—C5	38.8 (2)	C9—C10—C11—C12	1.1 (5)
N2^i^—Cu1—N1—C5	−123.9 (2)	C9—C10—C11—C13	−176.4 (3)
O4^ii^—Cu1—N1—C5	138.9 (2)	C10—C11—C12—C7	0.1 (4)
O1—Cu1—N1—C1	123.0 (5)	C13—C11—C12—C7	177.4 (3)
O5—Cu1—N1—C1	−147.0 (2)	C8—C7—C12—C11	−1.6 (4)
N2^i^—Cu1—N1—C1	50.3 (2)	C6—C7—C12—C11	−179.6 (3)
O4^ii^—Cu1—N1—C1	−46.9 (2)	C14—N4—C13—O7	11.6 (5)
O5—Cu1—O1—S1	−2.5 (3)	C14—N4—C13—C11	−167.5 (3)
N1—Cu1—O1—S1	87.3 (6)	C12—C11—C13—O7	−167.7 (3)
N2^i^—Cu1—O1—S1	160.7 (3)	C10—C11—C13—O7	9.7 (5)
O4^ii^—Cu1—O1—S1	−103.0 (3)	C12—C11—C13—N4	11.4 (5)
C5—N1—C1—C2	2.9 (4)	C10—C11—C13—N4	−171.3 (3)
Cu1—N1—C1—C2	−171.7 (2)	C13—N4—C14—C15	−168.0 (3)
N1—C1—C2—C3	−0.7 (5)	C13—N4—C14—C18	13.9 (5)
C6—N3—C3—C4	−9.6 (5)	C18—C14—C15—C16	0.2 (5)
C6—N3—C3—C2	173.1 (3)	N4—C14—C15—C16	−178.0 (3)
C1—C2—C3—N3	175.6 (3)	C17—N2—C16—C15	−2.2 (4)
C1—C2—C3—C4	−1.9 (4)	Cu1^i^—N2—C16—C15	−176.3 (2)
N3—C3—C4—C5	−175.1 (3)	C14—C15—C16—N2	1.7 (5)
C2—C3—C4—C5	2.2 (4)	C16—N2—C17—C18	0.9 (5)
C1—N1—C5—C4	−2.6 (4)	Cu1^i^—N2—C17—C18	175.4 (2)
Cu1—N1—C5—C4	171.9 (2)	N2—C17—C18—C14	0.9 (5)
C3—C4—C5—N1	0.1 (5)	C15—C14—C18—C17	−1.5 (5)
C3—N3—C6—O6	−9.8 (5)	N4—C14—C18—C17	176.6 (3)
C3—N3—C6—C7	170.9 (3)	C20—N5—C19—O8	−173.9 (5)
O6—C6—C7—C12	150.7 (3)	C21—N5—C19—O8	4.0 (6)
N3—C6—C7—C12	−30.1 (4)	Cu1^ii^—O4—S1—O3	−161.85 (15)
O6—C6—C7—C8	−27.4 (4)	Cu1^ii^—O4—S1—O2	76.27 (18)
N3—C6—C7—C8	151.8 (3)	Cu1^ii^—O4—S1—O1	−43.6 (2)
C12—C7—C8—C9	1.9 (5)	Cu1—O1—S1—O4	125.2 (3)
C6—C7—C8—C9	180.0 (3)	Cu1—O1—S1—O3	−114.4 (3)
C7—C8—C9—C10	−0.7 (5)	Cu1—O1—S1—O2	4.8 (3)

Symmetry codes: (i) −*x*, −*y*+2, −*z*; (ii) −*x*+1, −*y*+3, −*z*.

### Hydrogen-bond geometry (Å, º)

**Table:**

*D*—H···*A*	*D*—H	H···*A*	*D*···*A*	*D*—H···*A*
N3—H3*A*···O2^iii^	0.86 (3)	2.03 (3)	2.867 (3)	164 (3)
N4—H4*A*···O3^iii^	0.87 (3)	2.25 (3)	3.103 (4)	168 (3)
O5—H5*A*···O8^iv^	0.82 (3)	1.81 (3)	2.626 (4)	179 (4)
O5—H5*B*···O2	0.81 (3)	1.90 (3)	2.684 (3)	164 (4)
C4—H4···O8	0.93	2.45	3.325 (4)	157
C18—H18···O6^v^	0.93	2.55	3.277 (4)	136
C19—H19···O4^vi^	0.93	2.47	3.286 (4)	146
C20—H20*C*···O3^vii^	0.96	2.58	3.226 (6)	125

Symmetry codes: (iii) *x*, *y*−1, *z*; (iv) −*x*+1, −*y*+2, −*z*+1; (v) −*x*, −*y*+1, −*z*+1; (vi) *x*, *y*−1, *z*+1; (vii) −*x*, −*y*+3, −*z*+1.
